# Gastrocnemius Myoelectric Control of a Robotic Hip Exoskeleton Can Reduce the User's Lower-Limb Muscle Activities at Push Off

**DOI:** 10.3389/fnins.2018.00071

**Published:** 2018-02-14

**Authors:** Lorenzo Grazi, Simona Crea, Andrea Parri, Raffaele Molino Lova, Silvestro Micera, Nicola Vitiello

**Affiliations:** ^1^The BioRobotics Institute, Scuola Superiore Sant'Anna, Pisa, Italy; ^2^Fondazione Don Carlo Gnocchi, Firenze, Italy; ^3^Bertarelli Foundation Chair in Translation Neuroengineering, Center for Neuroprosthetics and Institute of Bioengineering, School of Engineering, École Polytechnique Fédérale de Lausanne, Lausanne, Switzerland

**Keywords:** exoskeleton, EMG control, gait, hip orthosis, walking assistance

## Abstract

We present a novel assistive control strategy for a robotic hip exoskeleton for assisting hip flexion/extension, based on a proportional Electromyography (EMG) strategy. The novelty of the proposed controller relies on the use of the Gastrocnemius Medialis (GM) EMG signal instead of a hip flexor muscle, to control the hip flexion torque. This strategy has two main advantages: first, avoiding the placement of the EMG electrodes at the human–robot interface can reduce discomfort issues for the user and motion artifacts of the recorded signals; second, using a powerful signal for control, such as the GM, could improve the reliability of the control system. The control strategy has been tested on eight healthy subjects, walking with the robotic hip exoskeleton on the treadmill. We evaluated the controller performance and the effect of the assistance on muscle activities. The tuning of the assistance timing in the controller was subject dependent and varied across subjects. Two muscles could benefit more from the assistive strategy, namely the Rectus Femoris (directly assisted) and the Tibialis Anterior (indirectly assisted). A significant correlation was found between the timing of the delivered assistance (i.e., synchronism with the biological hip torque), and reduction of the hip flexors muscular activity during walking; instead, no significant correlations were found for peak torque and peak power. Results suggest that the timing of the assistance is the most significant parameter influencing the effectiveness of the control strategy. The findings of this work could be important for future studies aimed at developing assistive strategies for walking assistance exoskeletons.

## Introduction

Over the last decades, several lower-limb exoskeletons have been developed for gait assistance of people with pathological gait patterns or for augmenting human performance of healthy individuals (Pons et al., 2008). Many control strategies have been proposed and tested, with different approaches depending mostly on the residual movement capabilities of the target end-users (Yan et al., 2015). The control strategies developed for people without movement capabilities (e.g., complete spinal cord injury patients) aim to let the person stand and restore walking by means of predefined trajectory tracking of the lower-limb active joints (http://berkeleybionics.com; http://www.indego.com; http://rewalk.com; Wang et al., 2014). When people have residual lower-limb movement capabilities, the exoskeleton controller should be able to understand the user's intended movement, synchronize with the periodicity of the gait pattern and provide additional assistance in specific phases of the gait cycle. In this case, developing a control strategy that effectively interprets the information gathered from the sensors and delivers the necessary mechanical power to the user at the right time turns out fundamental for the exoskeleton to supply effective and natural assistance (Yan et al., 2015). In addition, the control strategy of a gait assistive device should be intuitive, in order to avoid burdening the user with an additional cognitive effort (Tucker et al., 2015).

A widely investigated assistive strategy for robotic exoskeletons consists of measuring the activation of the muscles involved in the movement by means of electromyographic (EMG) signals and applying an assistive torque proportional to the linear envelope (LE) of the EMGs, coherently with their agonist/antagonist action (Kiguchi and Imada, 2009; Kinnaird and Ferris, 2009; Sawicki and Ferris, 2009; Kao et al., 2010). Many examples can be found in the state of the art: HAL (http://www.cyberdyne.jp; Kawamoto et al., 2003), AFO (Kao et al., 2010), and KAFO (Sawicki and Ferris, 2009) use surface EMG signals to control the joint torques. In these cases, the main goal of the assistance is typically to restore a more efficient (physiological) gait pattern, that reduces the energy cost of walking (Ferris et al., 2008; Pons et al., 2008; Sawicki and Ferris, 2009) or muscular activation (Lenzi et al., 2013; Jackson and Collins, 2015).

Despite the intuitiveness of EMG proportional controllers, differently from AFOs or KAFOs, the development of a reliable EMG-based controller for a hip exoskeleton is challenging for two main reasons: first, the thigh cuffs that connect the device to the human might cover most of the thigh surface and the placement of electrodes under the cuffs may cause motion artifacts and discomfort to the user; second, in healthy subjects but also patients with muscle weakness of hip muscle groups (Lewis and Sahrmann, 2006), thigh muscle signals are not as powerful as ankle plantarflexors during walking, thus the detection of the onset of muscle activation might require complex signal processing techniques in real time.

To overcome these limitations, in this study we propose and validate a new myoelectric control strategy based on an ankle plantar-flexor muscle signal, i.e., the Gastrocnemius Medialis (GM) to control the flexion torque of an active pelvis orthosis (APO). We used the GM signal to control the hip flexion torque because its activation is easier to be collected compared to hip flexor muscle, i.e., the Rectus Femoris (RF) (Winter, 1990; Annaswamy et al., 1999) and it activates synergistically to the hip flexors in the late stance phase to accomplish swing initiation and forward progression (Winter, 1990; Mueller et al., 1994; Zajac et al., 2003).

The presented algorithm is a modified version of the one presented in Grazi et al. (2015). Compared to our previous work, in this study we included the possibility to slightly adjust the timing of the assistive torque based on the user's perception, in order to improve the human-robot synchrony.

Along with the description of the control strategy, we present the results of preliminary tests with eight healthy subjects, walking on the treadmill with the APO providing three levels of assistance, i.e., optimal, low, and high, customized on each subject. We assessed the performance of the GM EMG-based proportional controller during walking, by evaluating the synchrony of the delivered hip assistance with the hip flexors muscular activity and by assessing the effects of the assistance on lower-limb muscles activity.

## Materials and methods

### Experimental apparatus

The experimental apparatus comprises three modules: (i) the APO (Giovacchini et al., 2015), (ii) a pair of shoes equipped with pressure-sensitive insoles (Crea et al., 2014), and (iii) a commercial EMG recording system.

#### APO

**Figure 1:**
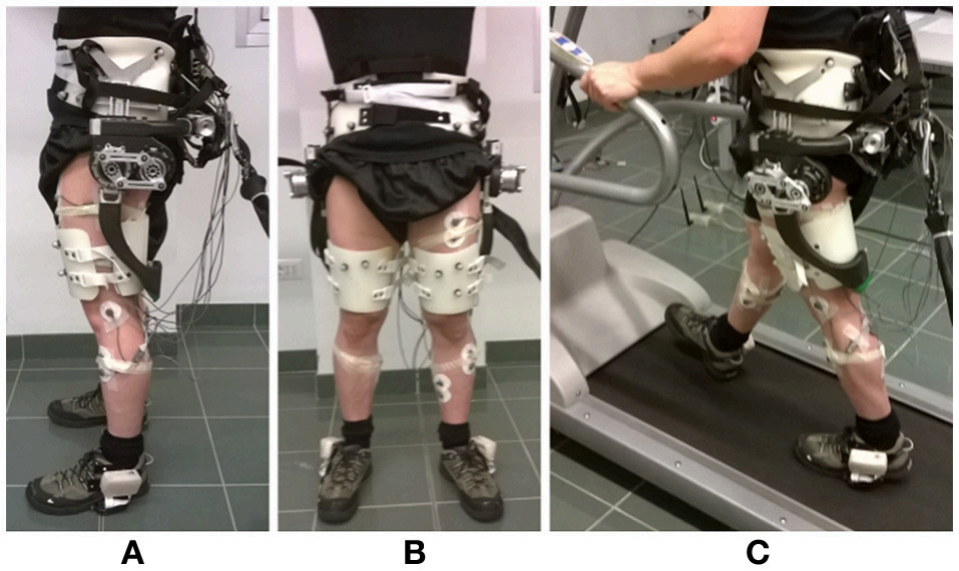
Overview of the Active Pelvis Orthosis. **(A)** Lateral view. **(B)** Frontal view. **(C)** During treadmill walking. Instrumented shoes and EMG electrodes are also shown.

The APO is a lightweight lower-limb exoskeleton, conceived for assisting the hip flexion-extension movement (Figure 1). A horizontal C-shaped frame, surrounding the user's hips, and the back of the pelvis interfaces with the user's trunk by means of three orthotic shells (two lateral and one rear) and carries two actuation units, one for each hip flexion-extension joint, mounted on the lateral arms. The actuation unit employs a SEA architecture (Pratt and Williamson, 1995) and can provide torque up to 35 Nm. The actuated axes drive two carbon-fiber links molded with a shape sweeping from the lateral to the back side of the thigh. Thigh links are also endowed with a passive rotational Degrees of freedom (DOF) for abduction-adduction to provide a comfortable interaction and do not hinder the user's movement while walking. The control and power supply units of the APO are remotely located in order to reduce the load carried by the user.

The APO control system runs at 1 kHz on a real-time controller, a cRIO-9082 (National Instruments, TX, USA), which is endowed with a 1.33 GHz dual-core processor running a real-time operating system and a field programmable gate array (FPGA) processor Spartan-6 LX150.

#### Instrumented shoes with pressure sensors

A pair of shoes was equipped with pressure-sensitive insoles for the measurement of the plantar pressure. Insoles consist of a matrix of pressure sensors and an electronic board endowed with Bluetooth® modules, for communication with external devices. Pressure signals were acquired and used to online extract the position of the plantar center of pressure in the longitudinal direction (CoP) and vertical ground reaction force (vGRF) (Crea et al., 2014).

Insoles were successfully used in other online applications, such as for providing lower-limb amputees with sensory feedback related to discrete gait events (Crea et al., 2015, 2017), to control an active prosthesis during different locomotion activities (Ambrozic et al., 2014) and to control an active lower-limb exoskeleton (Yuan et al., 2015).

#### EMG recording system

EMG signals were collected using pre-gelled bipolar Ag/AgCl surface electrodes (Pirrone & Co., Milan, Italy) and recorded by means of a TeleMyo 2400R EMG recording system (Noraxon Inc., AZ, USA). EMG signals were sampled at 1.5 kHz and low-pass filtered at 500 Hz by the EMG recording system. These biosignals are used both as feeding signals to the controller and to record muscles activity patterns during walking.

#### System integration

**Figure 2:**
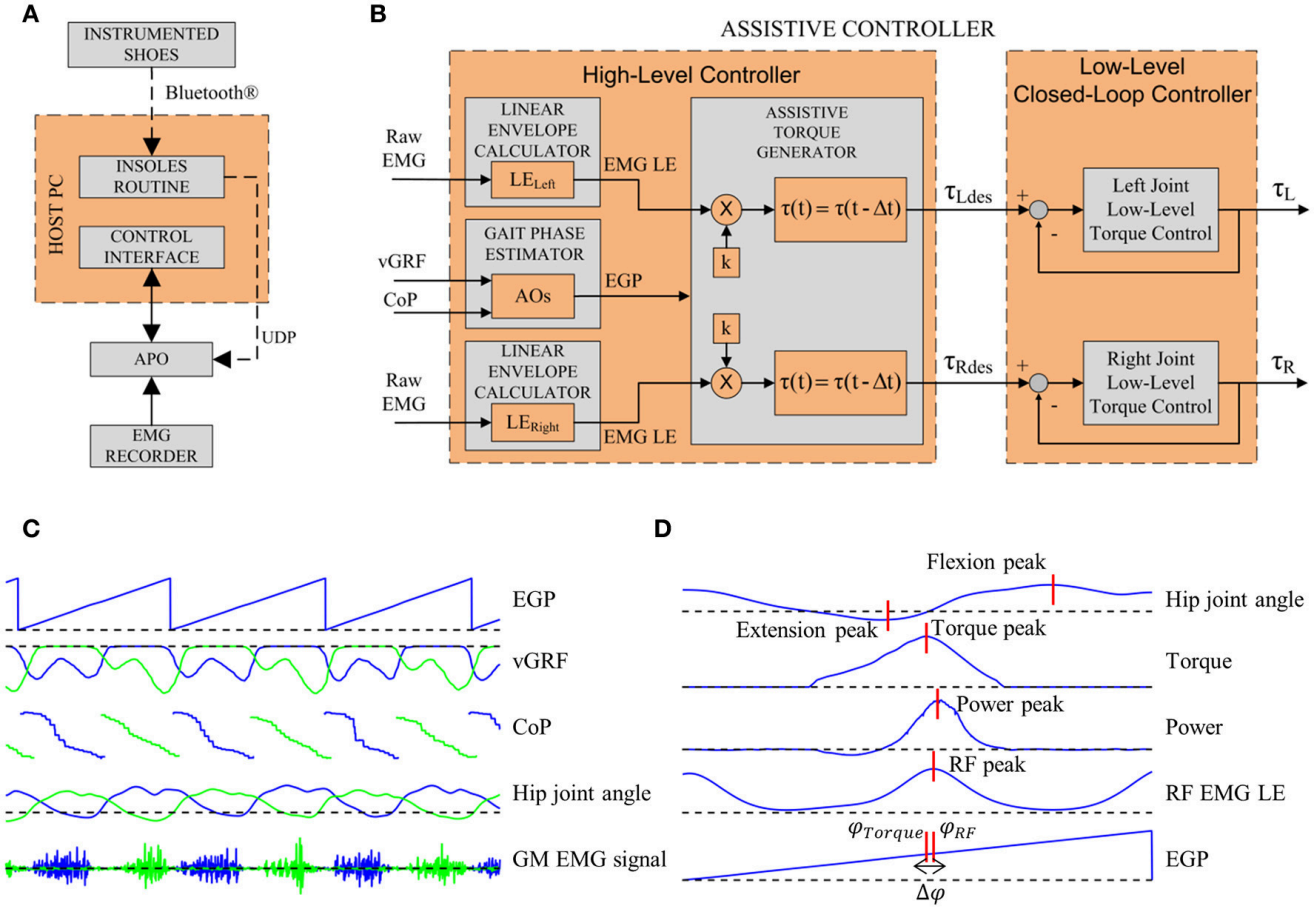
**(A)** Sub-modules of the experimental apparatus. **(B)** Architecture of the assistive controller. The inputs for the high-level control layer are the left vGRF, the CoP and the raw EMG signals of left and right GM muscles. The Gait Phase Estimator block computes the EGP. The Assistive Torque Generator block computes the LE. Each LE is then multiplied by *k* and delayed by Δ*t*. The outputs of the high-level control layer are the torque reference control signals for the low-level closed-loop controller. **(C)** EGP, vGRF, CoP, hip joint angle, and GM EMG signals for a few steps. Blue lines represent data from the left side, green lines represent data from the right side. **(D)** Parameters extracted.

The data gathered from the three modules were integrated into the control system as depicted in Figure 2A.

Data measured by the instrumented shoes were sent to a host PC through a Bluetooth® connection, read through a dedicated LabVIEW 2013 routine and, from the host PC, sent to the APO control board by means of a UDP connection.

The EMG recorder analog outputs were connected to the APO control board, i.e., the analog input channels of the cRIO-9082 FPGA. Data were acquired at 1 kHz and processed in real-time (Figure 2C).

Data were visualized on the host PC running a real-time LabVIEW 2013 Graphical User Interface (GUI). The GUI allowed also to set the parameters of the controller, and to save data for offline analysis.

### APO control architecture

The control system is based on a two-layer hierarchical architecture (Figure 2B): the low-level layer, which implements the torque control loop in order to set the current to the motor drivers, and the high-level layer, which implements the assistive control strategy.

*Low-level control*. The low-level torque control is designed to manage the actuators in order to follow a desired torque value. The closed-loop controller is a two-pole-two-zero compensator operating on the error between the desired (τ_*des*_) and actual (τ) torques. More details are reported in (Giovacchini et al., 2015).

*High-level control*. The high-level control relies on a two-step algorithm: the first step allows the estimation of the gait phase and the second step allows the calculation of the desired assistive torque.

#### Gait phase estimator

This block aims at estimating the current gait phase in real time by means of adaptive oscillators (AOs) (Ronsse et al., 2011, 2013). The vGRF signals from the sensorized insoles are the inputs for the AOs which, in turn, are used to estimate the current gait phase (EGP); the CoP information allows detecting the heel strike (HS) event and reset the phase given by the AOs (Yan et al., 2016). The APO control architecture using AOs and the HS event to reset the phase has been presented and validated in the work by Ruiz Garate et al. (2016). The computed gait phase ranges between 0 and 2π rad, corresponding to [0 ÷ 100] % of the stride period (the 0% of the stride corresponding to the HS and the 100% to the subsequent HS of the same foot). The EGP is computed unilaterally for the left leg and the assumption of gait symmetry is considered.

#### Linear envelope calculator

The EMG raw signal is high-pass filtered (second-order Butterworth, cut-off frequency: 20 Hz), rectified and then low-pass filtered (second-order Butterworth, cut-off frequency: 3 Hz) to obtain a LE of the EMG signal (Kinnaird and Ferris, 2009).

#### Assistive torque generator

The computed GM LE and EGP are used to calculate the assistive torque. The assistive torque is calculated when the EGP ranges between 20 and 60%: indeed, according to Winter's data (Winter, 1990), this phase corresponds to the hip flexors and GM activity in the propulsion phase. Within the selected gait phase window, when the online LE overcomes a threshold (i.e., five times the standard deviation of the GM signal during resting, as suggested by Kinnaird and Ferris (2009) the LE is multiplied by a gain, *k*, to obtain the desired torque. The assistive torque could be delayed by a percentage of the gait cycle, *T*, based on the user's subjective perception of the assistance (1):

(1) $$\{\begin{array}{c} \tau (t)=k\cdot LE_{GM}(t-\Delta t)\\ \Delta t=\frac{2\pi \cdot T}{\left|Freq_{gait}\right|} \end{array}$$

where τ is the assistive torque, *t* is time, and *t* is the time delay corresponding to *T. t* is a function of the gait frequency *Freq_gait_*–computed by the AOs—and *T. T* could be set up to 10%, according to the user's perception of a comfortable timing for assistance.

#### Experimental procedures

Upon arrival, the participant was informed about the study and asked to sign the written informed consent. Then s/he was asked to wear short sport pants, to allow easy placement of EMG electrodes, and wear the instrumented shoes.

The EMG electrodes were placed on the subject's left limb according to SENIAM recommendations (Hermens et al., 2000). The following muscles signals were recorded: Tibialis Anterior (TA), Vastus Lateralis (VL), Soleus (SOL), RF, Biceps Femoris (BF), Semitendinosus (ST), and GM. GM was recorded on the right and left limbs since it was necessary for generating the assistive torque.

Before wearing the APO, the subject was first requested to sit for 30 s, in order to record the EMG baseline signals and calculate the threshold for LE, and then to walk on the treadmill for 3 min at self-selected speed, in order to record the initial baseline (IBL) of the EMG activity during walking.

After the IBL was measured, the subject was asked to wear the exoskeleton, with the help of the experimenter.

A familiarization session was performed to tune the parameters for the assistive controller, i.e., the gain *k* and the phase delay *T* and let the subject familiarize with the assistance, walking at self-selected speed. Manual tuning was performed in two steps: first, *T* was set to 0% (i.e., the delivered torque was proportional to the GM EMG activation with no delay) and *k* was slowly increased from 0 until the maximum value for providing comfortable assistance; second, *T* was slightly modified in order to further increase the perception of comfort. Some iterations of the two steps could be repeated to fine tune the combination of the two parameters.

The testing session consisted of three trials with rests in between. Within each trial 3 min walking in assistive mode (AM) and 2 min walking in transparent mode (TM) were alternated. Within each single trial, three different levels of assistance were tested: the *k-*value set during the familiarization session (*k*_100_) was used to provide the “optimal” level of assistance (AM_100_); *k* was then scaled to 50% (*k*_50_) to provide the “low” level of assistance (AM_50_), and increased of 150% (*k*_150_) to provide the “high” level of assistance (AM_150_). The order of occurrence of the three assistive levels was randomized among trials and subjects.

After completing the three testing trials, subjects were requested to perform a 3 min walking session without the exoskeleton, in order to record the final EMG baseline (FBL).

### Participants

Eight healthy volunteers (5 male, age: 25 ± 1 years, height: 175.2 ± 7.2 cm, weight: 66.6 ± 4.6 kg) participated to the study.

Experimental trials were carried out at the premises of the rehabilitation center Fondazione Don Carlo Gnocchi (Florence, Italy). The experimental procedures were approved by the local Ethical Committee and carried out in accordance with the Declaration of Helsinki.

### Data analysis

EMG and exoskeleton data were saved for offline analysis. Data were segmented into strides, using the gait phase estimated online. For each trial, strides of the last minute of each condition were used to extract the following gait parameters, as depicted in Figure 2D:

- Maximum hip flexion angle;
- Maximum hip extension angle;
- Delivered peak hip torque, τ_*peak*_;
- Gait phase at the torque peak, φ_*torque*_;
- Power peak.

The eight LE signals were normalized by the median value of the maxima values determined in each stride of the last minute of the IBL. Normalization was done to allow inter-subject comparison. Normalized LE signals were processed to extract the following EMG parameters:

- LE peak;
- Gait phase at the RF LE peak, φ_*RF*_;
- Gait phase at the GM LE peak, φ_*GM*_;
- LE peak coefficient of variation, CV.

For each stride we calculated the parameter Δφ as the difference between φ_*RF*_ and φ_*torque*_. Δφ was used to assess the synchronicity of the torque provided by the exoskeleton with the RF EMG activity. Median values and interquartile ranges of Δφ were extracted for each AM in each trial. For each stride we computed Δψ as the difference between φ_*RF*_ and φ_*GM*_. Δψ was computed for IBL and AM conditions, to quantify the phase difference among the muscles activation peak and assess the goodness of the tuning procedure for the parameter *T*. Moreover, across-subjects average of the coefficients of variation (CV) were computed for RF and GM peaks with the goal of assessing the variability of the control signal. CVs were calculated from IBL data.

### Statistics

Friedman test was used to check for across-condition differences of gait and EMG parameters and when appropriate Wilcoxon signed-rank test was used for *post-hoc* paired comparison. The significance level was set to 0.05.

Pearson correlation was computed to check for correlations between the percentage variation of the EMG RF signal and one of the following parameters: Δφ, peak torque and peak power values. When significant correlation was found (*p* < 0.05), robust regression was computed and data were checked to find possible outliers by evaluating the residuals of the regression; residuals were identified by applying bisquare weighting method.

Data analysis and statistics were performed using Matlab R2017a (The Mathworks, MA, USA). All statistics were considered significant for *p* < 0.05.

## Results

### Controller performance

The across-subjects averaged Δφ values did not show significant differences among conditions (*p* = 0.19, χ^2^ = 3.25, dof = 2): 8.5 ± 3.7 % for AM_50_, 7.7 ± 3.1 % for AM_100_, and 7.9 ± 4.2 % for AM_150_. Positive values of Δφ indicate that, within the stride, RF peaks occur later than the GM peaks. Values for each subject are reported in Table 1.

**Table 1:** Controller parameters. Median and interquartile ranges for Δφ and τ_*peak*_ for each subject along with *T* and *k*_100_ values.

**Subject**	***T* (%)**	***k_100_***	**AM**_**50**_		**AM**_**100**_		**AM**_**150**_	
			**Δφ (%)**	**τ_peak_ (N·m)**	**Δφ (%)**	**τ_peak_ (N·m)**	**Δφ (%)**	**τ_peak_ (N·m)**
1	6.5	130	10.7 (8.2–12.0)	3.00 (2.75–3.25)	8.0 (6.5–9.9)	6.40 (5.82–6.93)	7.7 (5.0–11.1)	8.44 (7.75–9.15)
2	7.5	70	6.0 (3.5–9.1)	2.44 (2.30–2.74)	5.5 (2.0–9.8)	5.77 (4.93–6.25)	5.5 (3.7–7.9)	10.22 (9.64–10.94)
3	4.5	90	10.4 (8.4–14.4)	2.58 (2.41–2.74)	10.2 (7.7–11.7)	4.69 (4.26–5.04)	15.7 (13.8–17.9)	6.86 (6.43–7.70)
4	6.0	80	13.9 (11.6–16.0)	2.53 (2.32–2.74)	10.8 (8.7–12.6)	6.28 (5.56–6.77)	11.2 (6.7–14.3)	9.84 (9.05–11.64)
5	6.5	140	9.7 (7.4–10.9)	3.55 (3.18–3.86)	9.0 (8.0–10.3)	7.55 (6.58–8.81)	9.0 (7.5–10.7)	10.39 (9.49–11.08)
6	1.5	110	10.0 (8.9–10.7)	4.08 (3.32–4.76)	10.8 (8.7–12.7)	6.75 (6.13–8.05)	8.0 (7.2–9.3)	11.84 (10.33–13.07)
7	9.0	50	2.4 (1.9–2.9)	3.35 (3.04–3.80)	2.5 (1.9–3.1)	5.74 (5.13–6.31)	3.0 (1.9–3.8)	9.39 (8.99–10.08)
8	6.5	65	4.8 (3.1–10.1)	2.35 (2.17–2.53)	4.6 (3.5–5.5)	5.08 (4.49–5.64)	3.3 (1.8–6.1)	7.46 (6.70–8.06)

No significant differences were observed in the across-subjects averaged Δψ-values among conditions (*p* = 0.82, χ^2^ = 0.9, dof = 3): for IBL 13.7 ± 6.6, 15.1 ± 3.0% for AM_50_, 14.9 ± 2.1% for AM_100_, and 15.0 ± 4.0% for AM_150_. Table 2 reports values for each subject.

**Table 2:** Δ**ψ** median values and interquartile ranges for each subject for the initial baseline and all assistive conditions.

**Subject**	**IBL**	**AM_50_**	**AM_100_**	**AM_150_**
	**Δψ (%)**	**Δψ (%)**	**Δψ (%)**	**Δψ (%)**
1	14.8 (14–16.3)	15.7 (13.4–17.6)	15.3 (13.5–16.5)	14.5 (12.2–16.9)
2	12.6 (11.8–13.5)	14.1 (12.7–15.8)	13.7 (10.3–16.1)	12.4 (11.4–15.2)
3	11.8 (10.8–12.8)	16.1 (13.8–20.9)	15.2 (6.7–17.6)	22.2 (19.8–24.5)
4	23.3 (20.8–25.4)	21.2 (19.4–23.1)	18.8 (16.7–21.5)	19.0 (14.8–21.9)
5	17 (16.2–19)	16.7 (15.4–17.5)	16.9 (15.7–18.5)	17.2 (15.6–18.7)
6	13.1 (7.4–18)	12.7 (11.8–13.3)	13.6 (11.9–15.4)	11.0 (9.8–12.1)
7	14.4 (13–16.8)	12.6 (12.0–13.3)	13.4 (12.6–14.4)	13.1 (12.3–14.1)
8	16.3 (14.4–19.5)	11.6 (9.9–14.0)	12.2 (11.1–13.3)	10.9 (10.0–14.4)

Goodness of the tuning procedure of the parameter *T* was evaluated by comparing *T* with Δψ (the difference between these two parameters is Δφ); as the goodness of the tuning procedure decreased, Δφ-values increased.

Finally, the CV of the RF peak was greater than the one corresponding to the GM (0.72 compared to 0.37).

### Gait adaptation to the assistance

#### Kinematics and dynamics

**Figure 3:**
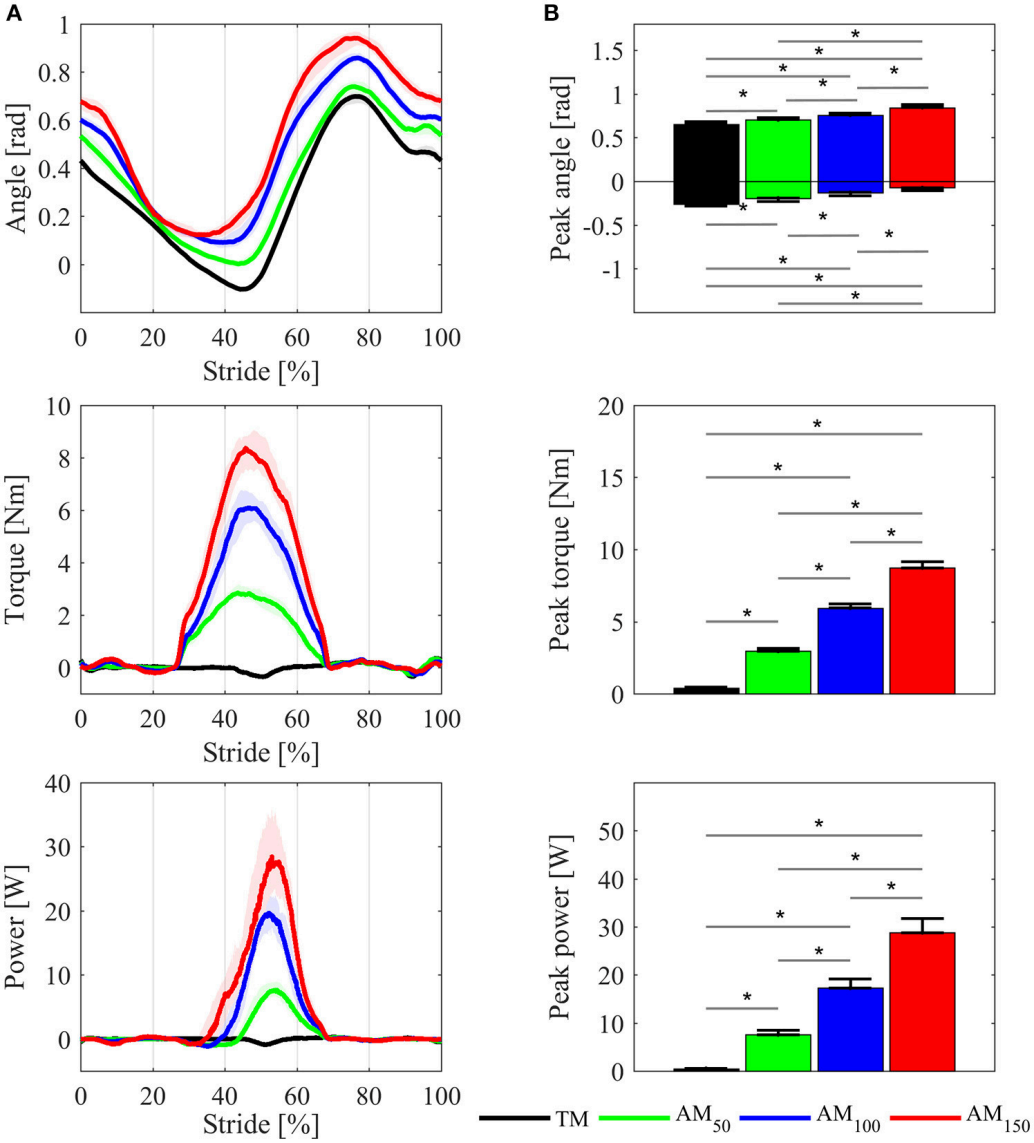
Comparisons of the APO kinematic and dynamic variables between TM, AM_50_, AM_100_, and AM_150_ conditions. **(A)** Hip joint angles, delivered torque and mechanical power profiles for one representative subject; variables are plotted as median values and interquartile range contours of left hip joint. **(B)** Maximum and minimum hip flexion angle, torque peak, and power peak, averaged over all subjects: mean values ± SE. Asterisks denote *p* < 0.05.

Kinematic and dynamic parameters are reported only for one side. Hip angle profiles for one representative subject are shown in Figure 3A. The across-subject average of the maximum and minimum peak angle values increased with higher levels of assistance (Figure 3B), and such variations were statistically significant (*p* < 0.05, χ^2^ = 13.63, dof = 3 for flexion peaks; *p* < 0.05, χ^2^ = 17.63, dof = 3 for extension peaks).

In the *k*_100_ condition, the delivered torque was on average around 6.0 ± 0.8 Nm. Under *k*_50_, with lower assistance, the torque peak was 3.0 ± 0.5 Nm, whereas in *k*_150_ condition with high assistance the torque peak was 9.0 ± 1.1 Nm. The mechanical power peak changed accordingly (17.0 ± 5.3 W for AM_100_, 7.0 ± 2.7 W for AM_50_, and 28.0 ± 8.3 W for AM_150_).

#### EMG analysis

**Figure 4:**
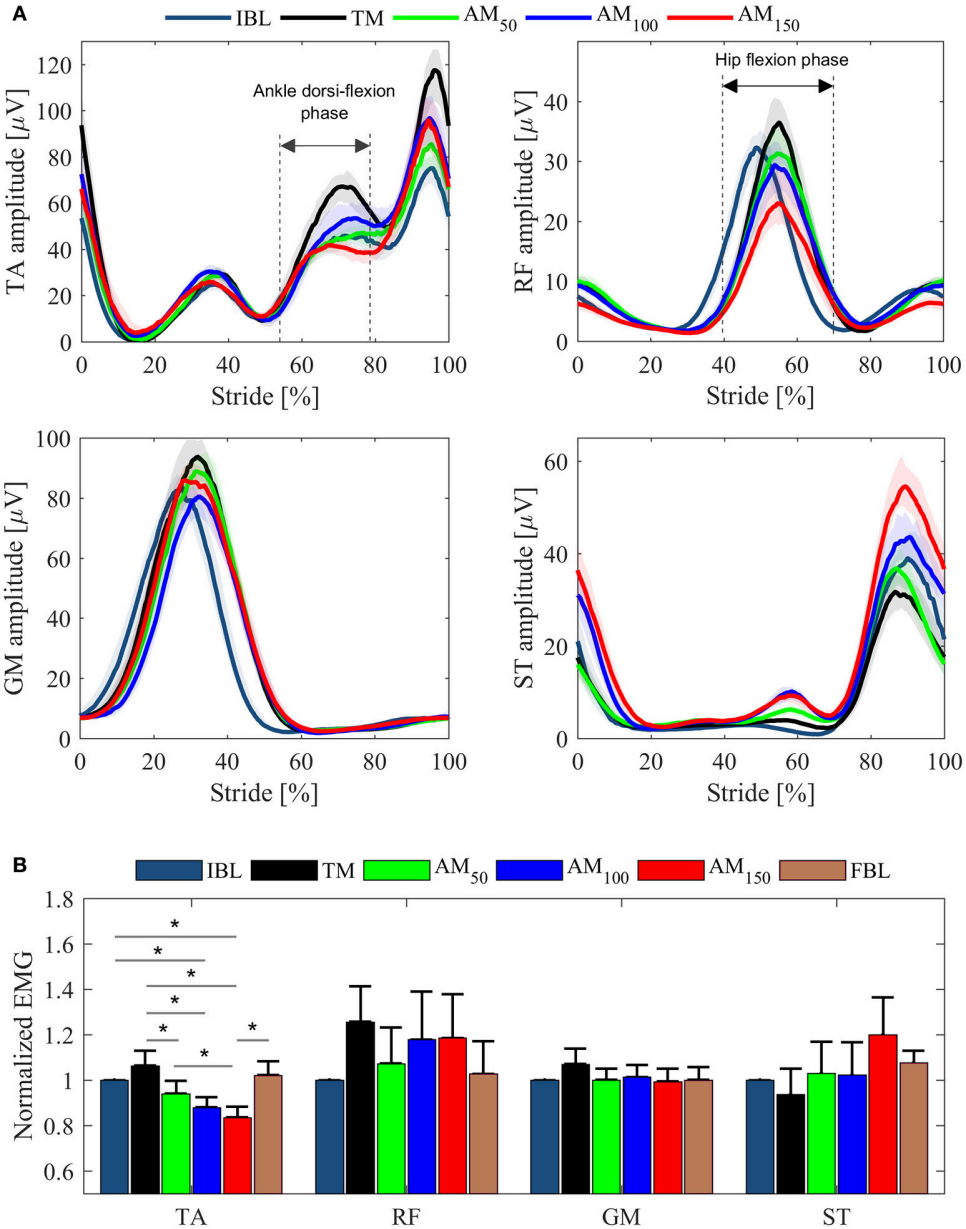
EMG data from left leg muscles (TA, RF, GM, ST) for the last minute of each tested condition. **(A)** EMG LE activation profiles for one representative subject. Profiles are shown as median values and interquartile range contours. **(B)** EMG peaks averaged over all subjects. Mean values ± SE are shown. Data are normalized for the IBL activation peak.

EMG pattern profiles for those muscles whose activity showed differences between conditions are shown in Figure 4A for one representative subject; normalized EMG data are depicted in Figure 4B as across-subjects average values. Note that only TA peaks reported significant EMG variations across conditions (*p* = 0.001, χ^2^ = 20.24, dof = 5). TA, RF and GM showed increased EMG activity when subjects walked under TM compared to IBL and FBL; on the contrary, EMG activity of ST reduced. Compared to the TM condition, TA, RF, and GM peaks, in the propulsion phase, decreased in all the AM conditions. For the ST, the muscle activity increased in all AM conditions with respect to TM. In general, baselines were similar before (IBL) and after (FBL) the experimental session, except for ST, which showed increased FBL EMG activity.

**Figure 5:**
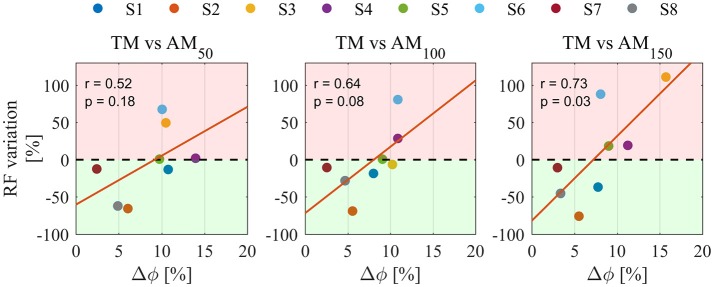
Pearson correlation analysis between Δφ and the percentage variation of RF EMGs for all assistance levels. Pearson correlation coefficient r and the level of significance p, are reported for each condition. Percentage variations are calculated for all AM conditions compared to TM. Colors are different participants. Red regions represent EMG activity increase, green regions represent EMG activity reduction. Orange lines indicate linear fit.

Figure 5 shows Pearson correlation analysis for RF EMG variations and Δφ. In the assistive conditions, correlation coefficients were found to increase with the assistance: *r* = 0.52 in AM_50_, *r* = 0.64 in AM_100_, *r* = 0.73 in AM_150_; moreover, also their significance increased with the assistance: no significance was found in AM_50_ (*p* = 0.18) and AM_100_ (*p* = 0.08), whereas significance was reached in AM_150_ (*p* < 0.05). For AM_150_, robust regression was performed and one outlier was found (corresponding to subject #6); once removed we obtained *r* = 0.84 (*p* < 0.02). No significant correlations were found for torque peaks (*p* = 0.09 in AM_50_, *p* = 0.22 in AM_100_, *p* = 0.97 in AM_150_) and power peaks (*p* = 0.8 in AM_50_, *p* = 0.25 in AM_100_, *p* = 0.19 in AM_150_).

## Discussion

Several studies showed that human Central nervous system (CNS) implements different strategies to adapt to external forces or disturbances, in order to ensure efficient locomotion (Kiguchi and Imada, 2009; Kinnaird and Ferris, 2009; Kao et al., 2010; Lewis and Ferris, 2011; Ronsse et al., 2011; Lenzi et al., 2013). In order to develop intuitive and effective control strategies for gait assistance and avoid muscle co-contractions, the assistive torque must be provided to the joint with perfect synchrony with the torque exerted by the biological limb, namely a coordination between the human and the exoskeleton must be established (Cenciarini and Dollar, 2011). To do so, state-of-the-art EMG controllers typically use the myoelectric signal measured from the muscles directly involved in the assisted action and provide an assistive torque proportional to the muscle activation (Kinnaird and Ferris, 2009; Sawicki and Ferris, 2009; Kao et al., 2010).

In the state of the art, many studies investigated the optimal assistance timing for ankle exoskeletons (Kinnaird and Ferris, 2009; Malcolm et al., 2013, 2015; Mooney et al., 2014; Collins et al., 2015; Jackson and Collins, 2015; Galle et al., 2017), whereas only few more recent studies have explored this issue for hip exoskeletons (Ding et al., 2016; Lee et al., 2017; Young et al., 2017b). Ding and colleagues investigated the effect of timing of hip extension assistance on biological joint power and metabolic cost of walking by means of a soft exosuit (Ding et al., 2016). The soft exosuit was used to deliver different hip assistive profiles, with the main goal of studying how the selection of the onset and the duration of the assistive torque can affect the metabolic cost of walking and found clear correlation between the two parameters. Similarly, Young and colleagues investigated the optimal timing to deliver the hip assistance through a hip exoskeleton and found that the subjective perception of the delivery timing did not correlate with the higher reduction in the metabolic cost (Young et al., 2017b); this result suggests that relying on this information would maybe improve the subjective perceived comfort of the user but not necessarily bring to optimal results in terms of metabolic consumption. Lee and colleagues explored the effects of different assistance timings on metabolic cost and gait parameters (e.g., step length and cadence) by means of an exoskeleton conceptually similar to the APO (Lee et al., 2017). In this study, researchers explored the effect of different timing offsets between the peaks of hip joint velocity and assistance power: results showed that smaller timing offsets led to higher reductions in the metabolic cost.

The novelty of the myoelectric controller proposed in this work is that it uses a calf myoelectric signal to provide a hip flexion torque during walking. During the propulsion phase, the GM together with the Achilles tendon, generates the ankle plantar-flexion torque to propel the gait, while the RF is responsible for generating the hip flexion torque (Townsend et al., 1978; Winter, 1990); in this phase, the two muscles typically show temporal synergic actions, i.e., they have almost synchronous activations (Zajac et al., 2003). Results of this study are in line with previous works. Indeed, we observed that the assistance was more effective when the assistance peak was synchronous with the RF EMG peak.

The analysis of EMG peaks revealed that two muscles can benefit more from the assistance when compared to the TM. While the reduction of the RF signal could be a direct effect of the provided hip flexion torque, the significant reduction of the TA activation can be explained by the induced increase of the foot clearance (due to the increased hip flexion angle). In addition, the resulting increased flexion and extension hip angles in all AM conditions were in line with previous studies with hip-assistive exoskeletons (Ronsse et al., 2011; Lenzi et al., 2013; Young et al., 2017b). However, while TA normalized EMGs showed reduced activation also with respect to the baselines, RF normalized EMGs resulted higher, which could be explained by the fact that the synchronism between assistance and hip flexors EMG activity was not enough to reduce muscles activations even below the baseline.

Furthermore, correlation analysis was performed to observe whether the timing parameter Δφ could affect the variation of the RF EMGs. Correlation was found between Δφ and RF EMG peak suggesting that lower Δφ could lead to higher EMG reductions. These results could support the use of the proposed control strategy and suggest, for future studies, its use to further investigate how different assistance timing could affect muscles activity. However, due to the small sample size of this study, it is difficult to draw definitive conclusions on the effect of assistance timing, although evidences from other studies support these results.

As in Young et al. (2017b), in our study we relied on the subjective perception during the tuning procedure of the assistance and adjusted the torque delivery timing. Similarly, our results show that, in some cases, the subjective perception lead to worse synchrony between the assistance and the user's movement. Subjects may not have been able to recognize whether the assistance was synchronous enough, but could have based their judgments on the perceived comfort and stability of the gait, as pointed out in similar study (Young et al., 2017b). Furthermore, due to the poor reliability of the subjective perception, in future studies *T* could be rather tuned based on the computation of Δψ during baseline or familiarization sessions; indeed, since Δψ did not change across conditions, this parameter could ensure a reliable tuning procedure. In this way we could try to minimize Δφ values and increase the synchrony between exoskeleton torque and hip flexors EMG activity.

Similar to other walking assistance exoskeletons (van Asseldonk et al., 2008; Ronsse et al., 2011; Winfree et al., 2011; Lenzi et al., 2012; Knaepen et al., 2014; Sylos-Labini et al., 2014) the APO “transparent mode” performance can be affected by inertia and friction, which worsen the capability of the controller to achieve null output impedance. Due to these reasons, muscular activity can increase when the exoskeletons are controlled in TM, which might be related to the resistive action of the exoskeleton on the user's movement (van Asseldonk et al., 2008; Ronsse et al., 2011; Winfree et al., 2011; Lenzi et al., 2012; Knaepen et al., 2014; Sylos-Labini et al., 2014). Similar to previous studies, our results showed that when the APO was controlled in TM, all subjects increased their RF muscular activation compared to IBL, which suggests the need to carefully consider inertia and friction compensation strategies to improve the performance in TM.

Finally, using the GM instead of the RF signal to assist the hip flexion resulted in three additional advantages. First, since the GM activation was higher than the RF activation (Winter, 1990), the signal for the control was easier to be processed online. Second, the GM signal did not suffer from motion artifacts, which, on the contrary, would have affected the RF muscle signal, due to the mechanical interaction and relative movements between the thigh cuffs and the electrodes (Young et al., 2017a). Furthermore, as reported by Winter's data (Winter, 1990), also in our data we observed that the inter-subject variability of the GM was considerably lower than the RF, thus a more reliable controller was developed.

## Limitations of the study and future perspectives

The goal of most robotic lower-limb exoskeletons is to reduce the metabolic cost of walking of the user (Ferris et al., 2008). Understanding how to reduce the metabolic energy expenditure of human locomotion through exoskeletons is still a complex challenge, and a huge amount of research has been done to answer this debated question (Ronsse et al., 2011; Malcolm et al., 2013, 2015; Mooney et al., 2014; Collins et al., 2015; Ding et al., 2016; Young et al., 2017b). Changes in muscles activation timing and amplitude during the utilization of lower-limb exoskeletons can be revealed by the analysis of the EMG (Ferris et al., 2008). Therefore, EMG may better reflect the changes in the metabolic energy demand with respect to, for example, the analysis of joint dynamics alone (Ferris et al., 2008). However, EMG only is still not sufficient to prove any metabolic cost reduction, thus dedicated experimental trials must be performed in future activities. Moreover, the controller presented in this study did not work with sufficient performance with all the tested subjects. The controller parameters (namely *T* and *k*) were tuned according to the user subjective perception and this might not be a reliable strategy to determine the most effective assistance profile (Young et al., 2017b); indeed, in particular the set values of *T* were much smaller than the baseline Δψ values, clearly suggesting that improvements of the tuning phase are necessary. Moreover, due to inter-subject variability, the implicit use of Winter's gait profiles as initial reference for identifying the range of the assistance timing could have introduced an additional limitation in the tuning procedure. Future developments of the control algorithm should consider automated calibration procedures to set the timing parameter. Future studies will be carried out to verify that the proposed GM-based controller improves the performance of a proportional EMG controller based on thigh muscles.

## Conclusion

In this work, a novel assistive control strategy for an APO was presented. The GM EMG signal is recorded to provide EMG-proportional assistive torque at the hip joints.

When the assistive torque profile was synchronous with the hip flexors muscular activity, the assistance resulted in reduced activation of the assisted muscles, i.e., the RF (directly assisted) and the TA (indirectly assisted), suggesting that the proposed strategy can induce the re-modulation of muscles activations and reduce the effort.

Future works will be aimed at improving the robustness of the proposed controller by means of an automatic procedure for parameters tuning in order to reduce the source of error and maximize the synchrony of torque delivery with the hip flexors activation. Future tests will also evaluate the effects of the assistance on the metabolic consumption.

## Ethics statement

This study was carried out in accordance with the recommendations of Fondazione Don Gnocchi Ethical Committee with written informed consent from all subjects. All subjects gave written informed consent in accordance with the Declaration of Helsinki. The protocol was approved by the Fondazione Don Gnocchi Ethical Committee.

## Author contributions

LG: Carried out the experimental activities and data analysis, participated in the design of the study, and drafted the manuscript; SC: Significantly contributed to the data analysis and drafted the manuscript; AP: Carried out the experimental activities and drafted the manuscript; RM: Participated in the design and supervised the study; SM and NV: Conceived the study, participated in the design, and coordination of the study; LG and SC: Equally contributed to this work. All authors approved the submitted version of the manuscript.

### Conflict of interest statement

SC, AP, SM, and NV have commercial interests in IUVO S.r.l., a spinoff company of Scuola Superiore Sant'Anna. Currently, part of the IP protecting the APO technology described in the paper has been licensed to IUVO S.r.l. for commercial exploitation. The authors confirm that this did not affect the analysis of the results. All other authors declare that the research was conducted in the absence of any commercial or financial relationships that could be construed as a potential conflict of interest.

## Abbreviations

AM: Assistive mode
AOs: Adaptive oscillators
APO: Active Pelvis Orthosis
BF: Biceps Femoris
CV: Coefficient of variation
CoP: Center of pressure
CNS: Central nervous system
DOF: Degrees of freedom
EGP: Estimated gait phase
EMG: Electromyography
FBL: Final baseline
FPGA: Field programmable gate array
GM: Gastrocnemius Medialis
HS: Heel strike
LE: Linear envelope
RF: Rectus Femoris
SEA: Series elastic architecture
SOL: Soleus
ST: Semitendinosus
TA: Tibialis Anterior
TM: Transparent mode
vGRF: Vertical ground reaction force
VL: Vastus Lateralis.
